# Amphibians with infectious disease increase their reproductive effort: evidence for the terminal investment hypothesis

**DOI:** 10.1098/rsob.150251

**Published:** 2016-06-29

**Authors:** Laura A. Brannelly, Rebecca Webb, Lee F. Skerratt, Lee Berger

**Affiliations:** 1 One Health Research Group, College of Public Health, Medical and Veterinary Sciences, James Cook University, Townsville, Queensland, Australia; 2 Faculty of Veterinary and Agricultural Sciences, University of Melbourne, Parkville, Victoria, Australia

**Keywords:** chytrid fungus, oogenesis, reproduction, spermatogenesis, terminal investment, wildlife disease

## Abstract

Mounting an immune response to fight disease is costly for an organism and can reduce investment in another life-history trait, such as reproduction. The terminal investment hypothesis predicts that an organism will increase reproductive effort when threatened by disease. The reproductive fitness of amphibians infected with the deadly fungal pathogen *Batrachochytrium dendrobatidis* (*Bd*) is largely unknown. In this study, we explored gametogenesis in two endangered and susceptible frog species, *Pseudophryne corroboree* and *Litoria verreauxii alpina.* Gametogenesis, both oogenesis and spermatogenesis, increased when animals were experimentally infected with *Bd*. In *P. corroboree*, infected males have thicker germinal epithelium, and a larger proportion of spermatocytes. In *L. v. alpina*, infected males had more spermatic cell bundles in total, and a larger proportion of spermatozoa bundles. In female *L. v. alpina*, ovaries and oviducts were larger in infected animals, and there were more cells present within the ovaries. Terminal investment has consequences for the evolution of disease resistance in declining species. If infected animals are increasing reproductive efforts and producing more offspring before succumbing to disease, it is possible that population-level selection for disease resistance will be minimized.

## 1. Introduction

Overall fitness of an individual is controlled and restricted by finite levels of energy allocation. Therefore, life-history trade-offs in energy allocation between physiological processes such as reproduction and fighting infectious disease are fundamental to fitness [1]. Mounting an immune response is costly, and to effectively fight infection, a trade-off of resources occurs [2]. Many studies have characterized infections that lead to a reduction in various reproductive measurements, like gametogenesis (gamete development), fertility and parental care [3]. Decreased gametogenesis occurred in the northern cricket frog after antigenic stimulation [4], in mice exposed to *Toxoplasma gondii* [5], in cattle with viral diarrhoea [6] and pneumonia [7], in dogs with canine leishmaniasis [8], and in humans with *Helicobacter pylori* infection [9] and AIDS [10,11]. Decreased overall fertility has been reported in the insect *Rhodnius prolixus* when infected with *Trypanosoma rangeli* [12], and decreased parental care effort has been observed in blue tits [13], and in house sparrows as they combat infection [14]. However, in other cases, there is no loss in reproductive effort; for example, gametogenesis is unaffected by asymptomatic HIV infection in humans [15]. Subclinical infections of *Cowdria ruminantium* in ewes did not affect female fertility [16], and rate of conception was unaffected in humans with Behçet's syndrome (a rare autoimmune disorder that causes inflammation of the blood vessels) [17].

Alternatively, disease can lead to an enhanced reproductive effort, in accordance with the terminal investment hypothesis. Organisms are expected to increase reproductive investment when reproductive value declines. For example, when an organism is unlikely to reproduce in the future, it is in its interest to invest much of its energy into the current progeny [18,19]. Terminal investment is well documented with senescence in iteroparous animals; the young prioritize self-maintenance, while the old prioritize reproduction [20,21].

In the face of infection, illness or immune challenge, animals can increase their reproductive investment, which is one outcome of terminal investment. Reproductive investment can be measured through efforts in mating, parental care and gametogenesis. For example, when male mealworms are immunocompromised or under stress, they produce more sex pheromone and so increase mating opportunities [22–24]. Parental efforts are increased in older male blue-footed boobies when an immune response is elicited [20]. In immune-challenged house sparrows, females increased their reproductive effort by laying replacement clutches more often than control animals [21], and in Arctic breeding common eiders, females that laid larger clutches had lower immune responses to avian cholera [25]. When snails are exposed to a trematode, they compensate by increasing egg laying soon after exposure, regardless of whether they become infected [26]. In trematode-infected limpets, sexual maturity is reached earlier and gonads are much larger [27]. Investing in reproduction rather than fighting disease can be important for organisms to pass on their genes, a tenet of evolutionary theory.

Little is known about how infection affects reproduction in amphibians. The amphibian chytrid fungus *Batrachochytrium dendrobatidis* (*Bd*) has caused amphibian declines globally [28]. *Bd* is an epidermal pathogen, which causes chytridiomycosis characterized by hyperkeratosis, electrolyte loss and ultimately death by cardiac arrest [29]. There has been much research devoted to understanding the immune system of amphibians and identifying resistance mechanisms in various species [30–32], but mitigating mortality through increased resistance is not the only mechanism by which a species can respond to infection. A shift in life history, such as reproductive fitness, can also explain population persistence in the face of large disease-induced mortality. To date, only two studies have explored reproductive fitness in animals with *Bd* infection: (i) infected *Rana (Lithobates) pipiens* males had larger testicular size with more mature sperm, which suggests that exposed animals invest in more spermatogenesis [33], and (ii) infected wild *Litoria rheocola* in good body condition were more likely to be found calling than uninfected males [34]. Female reproductive investment has not been explored in this system.

For this study, we explored gametogenesis in two critically endangered species to assess the impacts of disease. The alpine tree frog, *Litoria verreauxii alpina*, and the southern corroboree frog, *Pseudophryne corroboree*, are endangered amphibians endemic to the Australian alpine regions of New South Wales and Victoria. Both species are highly susceptible and have significantly declined due to chytridiomycosis, with *P. corroboree* now functionally extinct in the wild [35–38]. Infection prevalence is highest during the breeding season, but while *Bd* infection prevalence in *P. corroboree* was typically about 30% in a few extant populations over the last 10 years, in *L. v. alpina* infection levels reach 100%, causing near complete population turnover each year [39,40]. *Pseudophryne corroboree* are long-lived (more than 12 years) and late to sexually mature (approx. 3 years), and have low fecundity, producing approximately 30 eggs per clutch [41]. *Litoria v. alpina* historically reach sexual maturity at 2 years and aggregately breed [42].

Understanding the impact of *Bd* infection on the individual is needed to improve management efforts for both of these species. Since the introduction of *Bd*, individuals are maturing faster but disease resistance may not be evolving at a population level [37,40]. In an infection experiment with *L. v. alpina,* susceptibility in frogs sourced from long-exposed sites was not consistently lower than frogs from a naive population, even though susceptibility varied among individuals and clutches [37,43]. As this species can breed before succumbing to infection [39,40], the opportunity to evolve disease resistance is dampened [44]. Annual recruitment success, characterized by high population turnover, has become key to this species persisting [40]. As many amphibian species become infected during the breeding season [39,45–48], and the incubation period for severe chytridiomycosis can be up to about four months, this provides an opportunity for susceptible frogs to breed prior to death, and therefore produce susceptible offspring. Furthermore, if infected and susceptible animals are stimulated to spawn more offspring, as will occur with terminal investment, selection for resistance is even less likely to occur. Understanding how *Bd* affects reproduction as well as mortality may identify new options for conservation management.

In this study, we measured reproductive effort in *P. corroboree* and *L. v. alpina* experimentally infected with *Bd.* In males, we counted spermatogenesis stages, number of spermatic cysts and seminiferous tubule area as a proxy for reproductive effort [4,5,33]. In female *L. v. alpina*, we measured mass of the gonads, and counted developed eggs and total grossly visible cells inside the ovaries. The effects of disease on reproduction are rarely studied in wildlife, especially in amphibians, and this is one of the first to explore *Bd* and reproduction in multiple species and to include effects on females and males.

## 2. Material and methods

### 2.1. Animal husbandry

Southern corroboree frogs (*P. corroboree*) that were sexually mature and excess to breeding programmes were delivered to James Cook University from the Amphibian Research Centre. They had been captive raised and ranged in age from 5 to 8 years old. *Pseudophryne corroboree* reach sexual maturity at 4–6 years, and the oldest animal found in the wild was 9 years old, but this species is known to survive much longer in captivity [41]. Animals were housed individually in 300 × 195 × 205 mm terrarium with a damp and crumpled paper towel substrate, at a room temperature of 18–20°C. They were fed *ad libitum* three times weekly pinhead (5–10 mm) crickets (*Acheta domestica*). Animals were misted twice daily for 60 s with reverse osmosis water, and not artificial pond water because *P. corroboree* inhabit pristine habitats and are sensitive to water quality (M. S. McFadden 2014, personal communication). Temperature and humidity were monitored daily. Terraria were cleaned fortnightly by replacing the paper towel substrate.

*Litoria v. alpina* that were excess to a reintroduction trial were delivered to James Cook University from Taronga Zoo. They had been captive raised from wild collected egg masses in spring 2011 and ranged from 2 (for the male trial) to 3 years old (for the female trial) over the course of these experiments. In the wild, *L. v. alpina* survive only one breeding season before succumbing to *Bd*, but before the introduction of the pathogen, animals could survive up to 7 years of age [40,49]. They were housed individually under the same conditions as above, but with gravel substrate, which was replaced every three months.

Both *P. corroboree* and *L. v. alpina* breed seasonally following snow melt in the spring [36,39] after a few months of overwintering at low temperatures. In this study, animals were housed under consistent temperatures and daylight lengths that did not mirror breeding season regimes, and the gametogenesis observed represented activity outside peak breeding season. While animals were sampled at different times throughout the experiment (see data collection below), we do not expect confounding from any temporal variations.

Despite the experiments not being undertaken during peak breeding, *L. v. alpina* females were gravid at time of infection, and males were sexually mature, with observable nuptial pads and darkened throat patches. Secondary sexual characteristics are difficult to observe in *P. corroboree* because of their dark coloration. Males of both species were heard calling over the course of the experiments, but call details or secondary sexual characteristics were not measured.

### 2.2. Inoculation

Animals were allowed to acclimate to their new environment for 7 days. We used two different isolates and protocols for inoculating the animals of two different species. *Pseudophryne corroboree* males were inoculated with a known virulent isolate of *Bd* from New South Wales (AbercrombieR-L.booroologensis-2009-LB1, Passage number 11) in March 2013 [38,50]. *Bd* was harvested from agar and tryptone, gelatin hydrolysate, lactose (TGhL) Petri plates which had been incubated at 23°C for 5 days. Plates were flooded with 3 ml of artificial spring water for 10 min to allow zoospores to release from zoosporangia. Inoculum was poured off the plates and zoospores were counted using a haemocytometer. *Pseudophryne corroboree* males (*n* = 17) were inoculated with 1 × 10^6^ zoospores by applying 3 ml of inoculum dripped onto the venter over their individual 40 ml inoculation container. Animals were kept in these containers for 6 h, and then transferred back into their terraria. This method of inoculation has been successfully used for terrestrial amphibians [38,50,51].

*Litoria v. alpina* were inoculated using two different methods. *Litoria v. alpina* females were inoculated in the same manner as *P. corroboree* in February 2015 (*n* = 7), but *L. v. alpina* males were inoculated in February 2014 with a different *Bd* strain from the same region and isolated from clinically infected *L. v alpina* just prior to the start of this trial (WastePoint-L.v.alpina-2013-LB2, Passage number 1). This is the first experiment testing virulence of this strain of *Bd*. *Litoria v. alpina* males (*n* = 10) were inoculated with 5 × 10^5^ zoospores in 10 ml of inoculum dripped onto their venter and allowed to run off into their individual inoculation containers. The animals were held in inoculation containers for 24 h before returned to their individual terraria.

The change in methods was necessary due to the initial low proportion of infected *L. v. alpina* (see Results). To overcome this variation between the species, we used an isolate of *Bd* cultured from *L. v. alpina* in 2013, with a larger volume of inoculum—adapted from a successful method used in other hylid frogs [37,47,51]. We do not expect the differences in protocols to confound the results because gametogenesis was analysed in all frogs at a similar late stage of infection when effects of chytridiomycosis were similar*.*

*Bd* negative control animals were mock-inoculated using uninfected Petri plates (*P. corroboree n* = 10; *L. v. alpina* males *n* = 7, females *n* = 8).

### 2.3. Data collection

Animals were swabbed for *Bd* presence (see below), weighed to the nearest 0.01 g and measured snout to venter (SVL) to the nearest 0.1 mm weekly. Animals were euthanized with an overdose of MS-222 when clinical signs of chytridiomycosis (inappetence, irregular skin sloughing, cutaneous erythema, splayed legs) were displayed and righting reflex was abolished in accordance with animal ethics. The experiment ended when the last infected animals succumbed to disease or 13 weeks after inoculation, which ever was earlier. All animals remaining (controls and one female *L. v. alpina* that survived but maintained a high infection load) were euthanized at the end of the experiment (days 90–100). Both left and right testes were dissected from animals within thirty minutes after euthanasia. Oviducts and ovaries of *L. v. alpina* were weighed separately to the nearest 0.001 g.

### 2.4. Testing for *Batrachochytrium dendrobatidis*

We tested for *Bd* infection by using qPCR on skin swabs [52]. The swabbing protocol is standardized by performing 45 strokes with a sterile rayon-tipped swab (MW-113, Medical Wire & Equipment) per animal: five on the middle of the venter, five on each side of the venter, five on each thigh and five on each limb. The swab was gently rotated during and between strokes to ensure the greatest amount of DNA was gathered on the swab. Genomic DNA is extracted from the swabs using the Prepman Ultra kit and 2 min of bead beating to break apart the fungal cell walls. The extract was analysed using quantitative PCR following Boyle *et al.* [52], with a positive and negative control, and a series of dilution standards—100, 10, 1 and 0.1 zoospore equivalents (ZE) made in house—to estimate zoospore load. After inoculation animals were tested once a week until succumbing to disease or the experiment ended.

### 2.5. Testis histology

Testes were fixed in 4% phosphate-buffered formaldehyde for at least 2 weeks; the left testis was sectioned in *P. corroboree* and the right testis was sectioned in *L. v. alpina.* Routine histological techniques were used to prepare the testes for light microscopy following standard methods [4]. Testes were dehydrated in a graded series of ethanol, cleared with xylene and embedded in paraffin. They were serially sectioned at 5 µm, affixed to glass slides and stained with haematoxylin followed by eosin counterstaining (H&E), and mounted with coverslips. Four randomly selected histosections were analysed for each animal.

All measurements were made using the computer software ImageJ to the nearest 0.0001 mm. The area of the histosection, area of the three largest circular seminiferous tubules and number of tubules per histosection were measured. In the largest circular tubules per histosection, maximum germinal epithelium was measured. In tubules with no luminal space, the germinal epithelium depth was estimated as half the diameter. It must be noted that these histological measurements produce a relative indication rather than accurate depths because it is not possible to ensure sections pass through the centre perpendicularly to the tubule.

**Figure 1.**
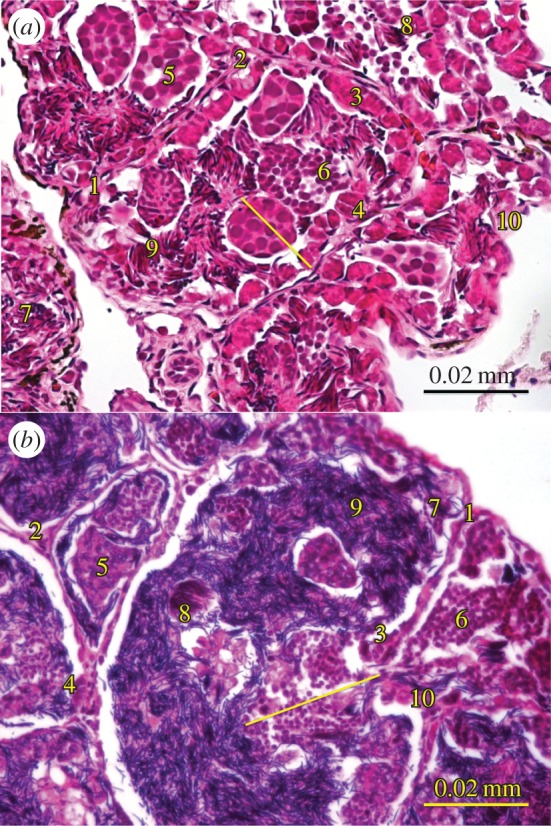
Spermatogenesis stages for (*a*) *Pseudophryne corroboree* and (*b*) *Litoria verreauxii alpina.* (1) Locular wall, (2) interlocular tissue, (3) primary spermatogonia, (4) secondary spermatogonia, (5) primary spermatocyte, (6) secondary spermatocyte, (7) primary spermatid, (8) secondary spermatid, (9) spermatozoa bundle and (10) Sertoli cells. Line indicates germinal epithelium depth. Magnification is 400×.

One field of view per histosection that included the largest seminiferous tubule was used to quantify spermatogenesis stages, with guidance from de Oliveira *et al.* [53] (figure 1). Within the seminiferous tubules, spermatogenesis stages can be quantified by number of cell layers in mammal and reptile testes [54,55], but in amphibians spermatogenetic cells occur in spermatic cysts rather than layers [53,56]; therefore, groups of cells were counted for each stage per field of view, and a spermatic cyst (a group of cells or cell bundle) typically has only one spermatogenesis stage [57]. The four main stages of spermatogenesis were counted: spermatogonia, spermatocytes (primary and secondary combined), spermatids (primary and secondary combined) and spermatozoa bundles (figure 1).

### 2.6. Female gonad assessment

The left ovary and oviducts were fixed in 4% buffered formaldehyde and sectioned following the procedure described above. These sections were analysed for pathology. To count eggs and total cells within the ovary, the right ovary was fixed in ethanol, the ovary membrane was separated and all cells within the ovary were counted grossly under 100× magnification for each animal. Cells within the ovary were grouped within two types: (i) developed ovum (with black yolk forming) and (ii) all other cells within the ovary, which includes all other stages of oogenesis such as oogonium and oocytes.

### 2.7. Statistical analysis

#### 2.7.1. *Batrachochytrium dendrobatidis* infection

Infection load was represented as median and interquartile range (IQR) of the ZE, which was calculated in SPSS v. 21. Infection loads are highly variable, but all animals included in the infected group had clinical chytridiomycosis, and died with high infection loads.

#### 2.7.2. Spermatogenesis

Distribution of all stages of spermatogenesis were analysed using Pearson's *χ*^2^-test on total cyst counts per spermatogenesis stage per individual. Significant results were further explored by calculating the mean proportion of each spermatogenesis stage cysts per individual in order to determine how each spermatogenesis stage differed between the uninfected and infected individuals. We compared these means of each spermatogenesis stage using independent two-tailed *t*-tests after normal distribution of the data was determined. Normal distribution of the data was determined using four measures: the distribution of the histogram, the ratio of mean to median, the ratio of mean to standard deviation and the Shapiro–Wilk test of normality.

Number of tubules per histosection, area of the histosections, area of the largest tubule per histosection and germinal epithelium depths were averaged for each individual and compared using independent *t*-tests, after normal distribution was determined. Only animals that succumbed to *Bd* during the experiment were included in the *Bd*+ group for analysis.

#### 2.7.3. Oogenesis

Number of cells within the ovaries and proportion of developed eggs compared with total cells within the ovary were analysed using Mann–Whitney non-parametric tests. Wet gonad mass was analysed for female *L. v. alpina* using the Mann–Whitney non-parametric test. Individual size was controlled for by analysing gonad mass/animal mass. Only animals that succumbed to *Bd* during the experiment were included in the *Bd*+ group for analysis.

All statistical analyses were conducted in SPSS (v. 21). Effect size was determined using Cohen's *d* statistic in Microsoft Excel.

## 3. Results

### 3.1. *Batrachochytrium dendrobatidis* infection

All uninfected control animals from both species, *P. corroboree* (*n* = 10) and *L. v. alpina* (*n* = 15), remained *Bd*− throughout the study. All 17 *Bd*+ *P. corroboree* died between days 29 and 81 (mean 45.94 ± 14.87 days) post-exposure. Median infection load at date of death was 124 317 ZE (IQR 126 851). Four of seven *L. v. alpina* females became infected with *Bd* after inoculation, and three died due to chytridiomycosis 58–60 days post-exposure, while one survived with a heavy infection until the end of the experiment (day 92). Median infection load in females at date of death or week 12 was 358 001 ZE (IQR 354 736). Six of the 10 inoculated *L. v. alpina* males developed chytridiomycosis and died between 39 and 63 days post-exposure (mean 52.67 ± 8.68 days). Infection load at date of death was 66 407 ZE (IQR 135 555).

### 3.2. Spermatogenesis

#### 3.2.1. Pseudophryne corroboree

**Figure 2.**
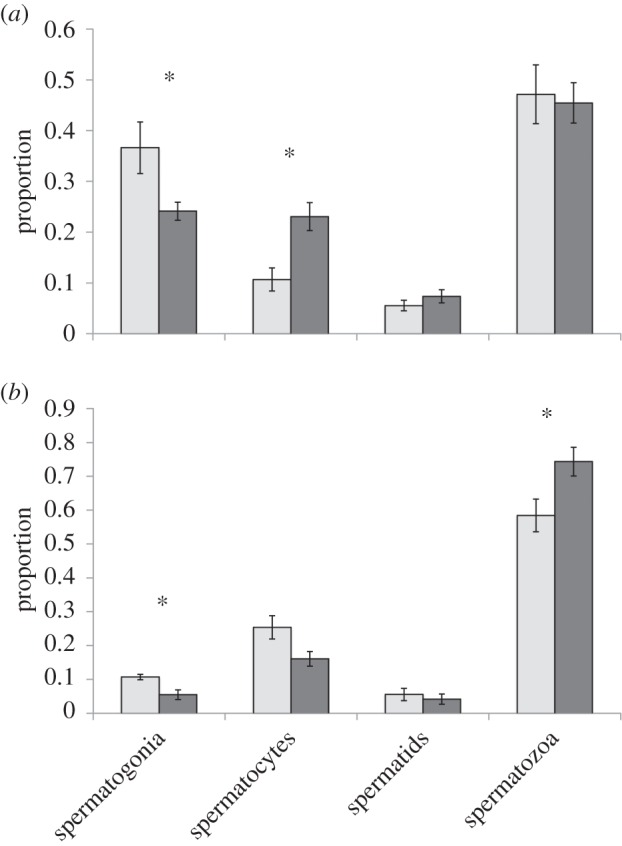
Spermatogenesis stage proportions found in the testes of (*a*) *Pseudophryne corroboree* (*Bd − n* = 10; *Bd* + *n* = 17) and (*b*) *Litoria verreauxii alpina* (*Bd − n* = 7, *Bd* + *n* = 6). Mean proportions of each spermatogenesis stage are graphed for *Bd*-infected (light grey boxes) and *Bd*-negative (dark grey boxes) individuals to represent the total cell bundles present in the testes. Error bars indicate standard error. Asterisks indicate a significant difference when *Bd*+ and *Bd−* were compared using a *t*-test. Only animals that succumbed to disease were included in the *Bd*+ group.

In male *P. corroboree*, overall proportions of spermatogenesis stages were significantly different between *Bd*+ and *Bd*− animals (*χ*^2^-test: 
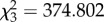
; *p* < 0.001; figure 2*a*). There was no difference in number of spermatogenesis-stage cysts per animal (*t*-test: *t*_24_ = 0.538, *p* = 0.596), but there were 116.9% more spermatocytes in the *Bd*+ animals (*t*-test: *t*_24_ = −2.813, *p* < 0.01, *d* = 1.32). The *Bd*− animals had a 32.9% higher proportion of spermatogonia (*t*-test, equal variances not assumed: *t*_8.76_ = 2.32, *p* = 0.046, *d* = 1.05).

*Bd*+ animals had a 46.5% larger germinal epithelium depth (*Bd*+ = 0.104 mm ± 0.035 mm; *Bd*− = 0.071 mm ± 0.035 mm; *t*-test: *t*_25_ = −2.556, *p* = 0.017, *d* = 0.94). There was no difference between *Bd*− and *Bd+* number of seminiferous tubules (*t*-test: *t*_24_ = −1.575, *p* = 0.128), area of histosections of testis (*t*-test: *t*_24_ = −0.34, *p* = 0.737) or area of tubules (*t*-test: *t*_24_ = 2.027, *p* = 0.053).

There was no difference in any measure between *Bd*+ animals that died before day 37 post-exposure and those that died between day 48 and 81 post-exposure.

#### 3.2.2. Litoria verreauxii alpina

In male *L. v. alpina*, overall proportions of spermatogenesis stages were significantly different between *Bd*− and *Bd+* animals (*χ*^2^-test: 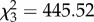, *p* < 0.001; figure 2*b*). There were 82.3% more spermatic cysts in the *Bd*+ animals (*t*-test: *t*_11_ = −3.746, *p* = 0.003, *d* = 2.02). There was 27.2% higher proportion of spermatozoa bundles in the *Bd*+ animals (*t*-test: *t*_11_ = −2.421, *p* = 0.034, *d* = 1.36), but 49.5% fewer spermatogonia (*t*-test: *t*_11_ = 3.302, *p* = 0.007, *d* = 1.85). There was no difference in the number of spermatocytes between the two groups (*t*-test: *t*_11_ = −0.532, *p* = 0.605).

No differences were found between the *Bd*+ and *Bd*− male *L. v. alpina* for germinal epithelium depth (*t*-test: *t*_11_ = 0.523, *p* = 0.612), seminiferous tubule number (*t*-test: *t*_11_ = −0.394, *p* = 0.702), seminiferous tubule size (*t*-test: *t*_11_ = 0.352, *p* = 0.731) or total histosection area (*t*-test: *t*_11_ = 0.393, *p* = 0.702).

### 3.3. Oogenesis

**Figure 3.**
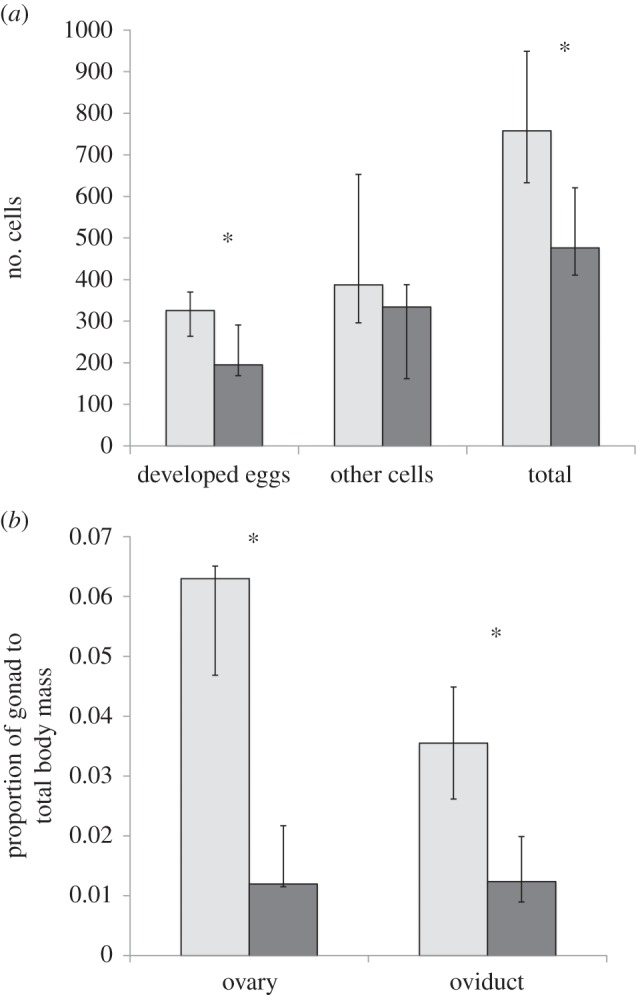
Oogenesis comparisons between infected and uninfected female *Litoria verreauxii alpina* (*Bd − n* = 7, *Bd* + *n* = 4). (*a*) Number or cells counted in the ovary per animal. (*b*) Gonad proportion of ovary and oviduct compared with the body mass of the individual. Central tendency is presented as the median of *Bd*-infected (light grey boxes) and *Bd*-negative (dark grey boxes) individuals. Error bars indicate interquartile range. Asterisks indicate a significant difference when *Bd*+ and *Bd−* were compared using a Mann–Whitney *U*-test. Only animals that succumbed to disease were included in the *Bd*+ group.

Counts of grossly visible eggs in female *L. v. alpina* revealed infected animals had 59.1% more cells inside the egg masses compared with uninfected (Mann–Whitney: *Z* = −2.084, *p* = 0.037) and 67.2% more developed eggs present in the ovaries (Mann–Whitney: *Z* = −2.079, *p* = 0.038), but there was no difference in proportion of developed eggs to other cell types within the masses between *Bd*+ and *Bd*− animals (Mann–Whitney: *Z* = 0, *p* = 1; figure 3*a*).

Ovaries of infected animals were 2.15× bigger as a proportion of body size than ovaries of uninfected animals (Mann–Whitney: *Z* = −2.548, *p* = 0.011). Oviducts of infected animals were 1.56× bigger as a proportion of body size than oviducts of uninfected animals (Mann–Whitney: *Z* = −2.717, *p* < 0.01; figure 3*b*).

#### 3.3.1. Ovary pathology

There was a wide range of stages of development of eggs within an individual and among individuals. Development ranged from early egg stages to fully developed and full-of-yolk platelets to atresia of developed eggs that were being reabsorbed. Oviducts looked normal. There were no obvious differences in the ovaries and oviducts between the infected and uninfected animals.

## 4. Discussion

Our study shows that more gametogenesis occurred in male and female frogs experimentally infected with the fungus *Bd.* Increased gametogenesis is a proxy for increased reproductive effort [4,5,7,33], which may result in more offspring. Mounting an immune response to fight disease represents an energy cost for the host. Some organisms may prioritize reproduction over investing in immunity, a hypothesis known as terminal investment. Terminal investment in species susceptible to the deadly amphibian chytrid fungus may have enabled populations to persist but have resulted in them not evolving disease resistance.

### 4.1. Spermatogenesis

In *P. corroboree* males, there were a greater proportion of spermatocytes and a deeper germinal epithelium, suggesting higher production of spermatozoa after infection. There were more spermatogenesis cell bundles and more spermatozoa bundles present in infected *L. v. alpina*, consistent with greater sperm production and storage. Higher proportions of spermatocytes and spermatozoa are correlated with higher reproductive success in other taxa [58], suggesting a similar pattern of increased reproduction in these frog species.

Both species had a lower proportion of spermatogonia in the *Bd*+ animals. Spermatogonia are the early stages of spermatogenesis, the stock stem cells [57]. When the spermatogonia cells divide, some become committed cells that differentiate into spermatozoa, while a subset remain stem cell spermatogonia. Therefore, the number of spermatogonia present in the testis does not change [59]. However, spermatocytes (the first stage of sperm development that undergo mitosis), spermatids (later stages where meiosis is undertaken) and spermatozoa bundles (the mature sperm cells) increase with more sperm production. Therefore, a lower proportion of spermatogonia is consistent with a phase of active spermatogenesis.

While timing of spermatogenesis varies among species and has never been explicitly studied in the species studied here, the entire process of spermatogenesis in amphibians can range from approximately 30 days to four months [57]. The long incubation period of chytridiomycosis as seen here of one or more months enables frogs to appear unaffected and in good body condition, only showing clinical signs in the last few days when severe disease manifests. Therefore, increased reproduction and even life-history shifts towards increased reproduction is feasible during the subclinical phase of chytridiomycosis.

### 4.2. Oogenesis

In the female *L. v. alpina*, the ovaries and oviducts were much larger in infected animals. In addition, there were more cells present in the ovaries of infected females, demonstrating a functional increase rather than pathological swelling. This finding is surprising because all females were gravid at the time of initial exposure, and oogenesis is more time intensive and energy consuming than spermatogenesis [60]. Oogenesis and female investment is more logistically difficult to quantify than male investment; therefore, it is less often studied. In female amphibians, exact length of time for the full oogenesis cycle is unknown, except that females appear to lay eggs either once or twice a year [57], suggesting that oogenesis is a much longer cycle than spermatogenesis. Therefore, we recommend further work in this area to test our initial findings, which were based on a few animals and breeding outside of the peak season.

### 4.3. Gametogenesis and *Batrachochytrium dendrobatidis*

Our results extend previous findings from two species suggesting frogs increase reproductive investment via mating displays or gametogenesis when infected with *Bd*. Wild *L. rheocola* males were found calling more often upon capture when infected with *Bd* [34], suggesting an increased mating effort, while *R. pipiens* had longer testes with a higher proportion of mature spermatozoa when experimentally infected with *Bd* [33], suggesting increased gamete production. Our study explored reproduction in both males and females by assessing gametogenesis and quantifying the differences in gamete production. This reproductive response in four frog species may be *Bd* specific, due to the immunosuppressive effects of the fungus, or due more generally to subclinical disease, because antigenic stimulation (a non-specific substitute for the immune response aspect of disease) in another frog species decreased reproductive investment [4].

We used histological measurements as a proxy for sperm production, but gamete viability, mating success, normal embryo development, offspring survival and overall reproductive fitness cannot be determined using this method. While spermatogenesis is often used as a proxy for reproductive investment [4,5,7,33], we do not know how the increases in gametogenesis that we observed translate into mating success or increased reproductive fecundity. Our study is the first step in understanding how disease impacts reproduction, but more research is needed to fully understand the phenomenon. At this stage, the mechanism of increased gametogenesis is not known, and could be a result of resource partitioning by the animal as per terminal investment, or a hormone-like chemical produced by the pathogen.

### 4.4. Population persistence

*Batrachochytrium dendrobatidis* infects over 600 amphibian species globally [28,61], but while many species are currently declining, some populations appear to be rebounding since the original epidemic [62,63]. Population rebound may point to natural selection for disease resistance or decreased virulence of the pathogen, as occurred after introduction of myxomatosis to rabbits [64]. Evolution of resistance should occur if animals preferentially breed after surviving exposure, and this may explain the pattern of recovery in some species where longevity is increasing (e.g. [62]) However, with *Bd* infection, some species may lack an effective innate immune response, and the adaptive immune response may be suppressed [65–67], and while different strains of *Bd* differ in virulence [47,68] there is no clear evidence of decreasing pathogen virulence over time [69]. Therefore, another mechanism of population-level persistence, such as increased reproduction, might explain the lack of widespread resistance within a population with endemic disease.

The terminal investment hypothesis refers to the trade-off between investing in one large but final reproductive event versus investing in survival and future breeding. Here we propose that a population threatened by chytridiomycosis adopts the terminal investment strategy, and that higher reproductive output (whether innate or stimulated by infection) will dampen the population-level evolution of disease resistance. For *L. v. alpina*, progeny survival has enabled populations to survive so far, but appears a precarious strategy as it is dependent on uninterrupted breeding seasons [40]. One generation of failed recruitment will lead to population extirpation.

*Pseudophryne corroboree* is a low-fecundity, long-lived species that declined gradually after *Bd* introduction. Even if there has been an increase in reproduction after infection it has not been enough for populations to survive and the species is now functionally extinct in the wild [70]. For both species, perhaps an increase in reproduction and resistance to infection would help avoid population extirpation and species extinction.

## 5. Conclusion

Our results suggest that increased reproductive investment might be more widespread than previously thought, adding amphibians and fungi to the list of hosts and pathogens that are involved in this response. Terminal investment of infected animals has consequences for conservation management of declining species. With an increase of infected and susceptible animals reproducing, population-level selection for disease resistance or tolerance is likely to be minimized. Artificial selection for resistance has been proposed as a management technique for mitigating *Bd* [71,72], but if natural selection in wild persisting populations has led to other outcomes such as increased reproduction associated with terminal investment, then which direction should interventions take? Management of the habitat [71] to support recruitment appears critical in the short term for the two species investigated here, but is also applicable to a broader range of species. However, a concurrent approach of understanding and promoting genes for innate resistance factors is also likely to be useful to increase individual longevity, and therefore population security.

## Supplementary Material

Data

## Data accessibility

The datasets supporting this article have been uploaded as part of the electronic supplementary material.
